# A multi-omics systems vaccinology resource to develop and test computational models of immunity

**DOI:** 10.1016/j.crmeth.2024.100731

**Published:** 2024-03-14

**Authors:** Pramod Shinde, Ferran Soldevila, Joaquin Reyna, Minori Aoki, Mikkel Rasmussen, Lisa Willemsen, Mari Kojima, Brendan Ha, Jason A. Greenbaum, James A. Overton, Hector Guzman-Orozco, Somayeh Nili, Shelby Orfield, Jeremy P. Gygi, Ricardo da Silva Antunes, Alessandro Sette, Barry Grant, Lars Rønn Olsen, Anna Konstorum, Leying Guan, Ferhat Ay, Steven H. Kleinstein, Bjoern Peters

**Affiliations:** 1 Center for Infectious Disease and Vaccine Research, La Jolla Institute for Immunology, La Jolla, CA, USA; 2 Bioinformatics and Systems Biology Graduate Program, University of California, San Diego, San Diego, CA, USA; 3 Department of Health Technology, Technical University of Denmark, Kongens Lyngby, Denmark; 4 Knocean Inc., 107 Quebec Avenue, Toronto, Ontario M6P 2T3, Canada; 5 Program in Computational Biology & Bioinformatics, Yale University, New Haven, CT, USA; 6 Department of Medicine, University of California, San Diego, San Diego, CA, USA; 7 Department of Molecular Biology, School of Biological Sciences, University of California, San Diego, La Jolla, CA, USA; 8 Department of Pathology, Yale University School of Medicine, New Haven, CT, USA; 9 Department of Biostatistics, Yale School of Public Health, New Haven, CT, USA

**Keywords:** Multi-omics integration, Systems vaccinology, Bordetella Pertussis, Prediction models, Contest, Vaccine response, Reproducibility

## Abstract

Systems vaccinology studies have identified factors affecting individual vaccine responses, but comparing these findings is challenging due to varying study designs. To address this lack of reproducibility, we established a community resource for comparing *Bordetella pertussis* booster responses and to host annual contests for predicting patients' vaccination outcomes. We report here on our experiences with the “dry-run” prediction contest. We found that, among 20+ models adopted from the literature, the most successful model predicting vaccination outcome was based on age alone. This confirms our concerns about the reproducibility of conclusions between different vaccinology studies. Further, we found that, for newly trained models, handling of baseline information on the target variables was crucial. Overall, multiple co-inertia analysis gave the best results of the tested modeling approaches. Our goal is to engage community in these prediction challenges by making data and models available and opening a public contest in August 2024.

## Highlights

• CMI-PB is a prospective challenge project for models of vaccine-induced immunity
• Dataset comprises multi-omics profiling pre- and post-vaccine against *B. pertussis*
• Test 20+ models pulled from the literature and variable prediction performance found
• Tailored multi-omics models and simple age-based models currently perform well

## Motivation

Several systems vaccinology studies have generated large datasets on the immune states of individuals before and after vaccination and have identified factors that drive differences in individual vaccine responses. However, it has been challenging to test how well conclusions from one study generalize across others given the differences in design. We aim to address this lack of reproducibility by establishing a community resource and engaging the research community through open prediction challenges that allow development and comparison of models that predict the immune response of human booster vaccinations for *Bordetella pertussis*.

## Graphical abstract

**Figure:**
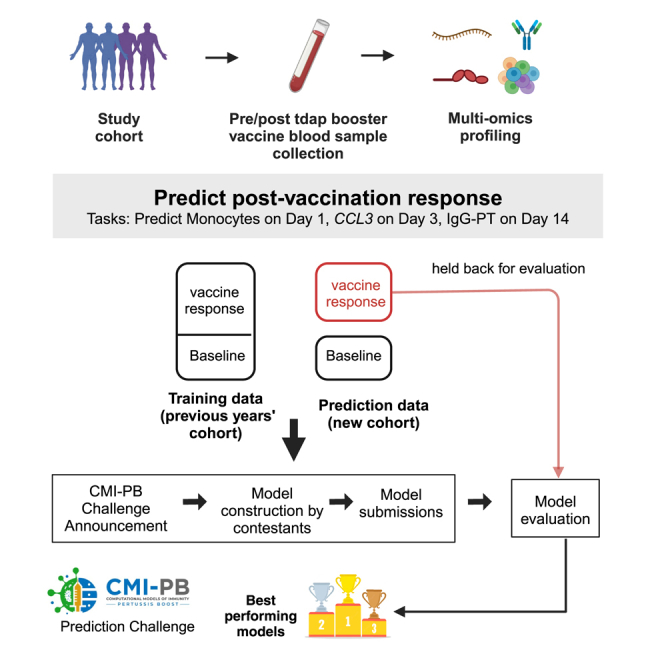

## Teaser

Shinde et al. establish a community resource to compare patients' vaccine responses and to host annual contests to predict vaccination outcomes. They find specifically trained multi-omics models and simple age-based models outperformed. They aim to engage the community with a public prediction contest starting August 2024.

## Introduction

The overall goal of our study is to provide a resource to develop and test computational models of vaccine-induced immunity. Our specific focus is on whooping cough, a vaccine-preventable, highly contagious respiratory infection caused by *Bordetella pertussis* that mainly affects infants.^1^ The development of the first pertussis vaccine was initiated in 1914 and became widely available in the 1940s when the whole-cell pertussis compounds (wP) vaccine was combined with diphtheria and tetanus toxins to make the DTwP vaccine.^2^^,^^3^^,^^4^ With the introduction of this vaccine, the number of reported whooping cough cases in the United States declined from approximately 200,000 a year in the pre-vaccine era to a low of 1,010 cases in 1976.^5^ Due to side effects reported with the use of wP vaccine, wP compounds in the DTwP vaccine were replaced with acellular pertussis (aP) antigens, leading to the development of a new and less reactogenic vaccine (DTaP) in 1991.^6^ Booster vaccines were similarly updated to include the acellular pertussis antigens (Tdap), which are routinely scheduled to be administered to teens and adults every 10 years.^7^

While the aP vaccines provided protection from whooping cough equivalent to that of wP vaccines in clinical trials covering the initial period after vaccination, questions have been raised about their long-term durability^8^^,^^9^ and protection against transmission.^10^^,^^11^ Specifically, an increase in pertussis outbreaks has been reported in various countries that have switched from wP to aP vaccines,^12^^,^^13^ including the United States (data available from Pertussis Cases by Year,^14^ accessed 15 May 2023). Many of these outbreaks occurred among children who only received aP vaccines. As a result, multiple studies about waning immunity post aP vaccination were conducted,^15^^,^^16^^,^^17^^,^^18^^,^^19^ including the characterization of differences in the immune response against aP and wP vaccines.^7^^,^^20^^,^^21^^,^^22^^,^^23^^,^^24^^,^^25^^,^^26^ Some studies, including our own,^7^^,^^23^^,^^24^ showed that there are long-lasting effects and differences in polarization and proliferation of T cell responses in adults originally vaccinated (primed) with aP vs. wP, despite subsequent Tdap booster vaccination.^20^^,^^21^ However, it remains unclear how this difference in immune responses is maintained over time between individuals primed with an aP vs. a wP vaccine.

To address these questions, our near-term goal is to determine how an individual responds to pertussis antigen re-encounter by characterizing the resulting cascade of events (i.e., recall memory response) and relating it to the pre-vaccination immune state. To achieve this, we apply a systems vaccinology approach that integrates different biological readouts such as transcriptomic, proteomic, and cytometric data to broadly define the immune state of an individual and to define changes in a pre- and post-vaccine setting. Subsequently, we create computational models connecting the pre-vaccination state of an individual to the final vaccination outcome after pertussis boost. Previous studies have identified pre-vaccination immune signatures that are associated with high antibody titers post vaccination in different vaccine settings, most prominently for influenza A vaccination, but none had been applied to pertussis booster vaccines before.^27^^,^^28^^,^^29^^,^^30^^,^^31^ Our long-term goal is to use a predictive understanding of pertussis booster responses to identify what differentiates aP from wP primed individuals and to determine the desirable characteristics of an elicited vaccine response.

A common challenge in developing computational models for biological applications is to objectively test their generalizability and predictive performance.^32^^,^^33^^,^^34^ This is especially challenging for systems vaccinology studies, as the design varies among studies. The multidimensional and heterogeneous nature of systems vaccinology data poses significant challenges for model development and validation. The presence of numerous features and a limited sample size further exacerbates the difficulties to conventional machine-learning (ML) and deep-learning methods. Overfitting is a crucial issue in a setting such as this, which is why testing any algorithm generated from the training data on a completely independent dataset (and new cohort) is so important. Integrating diverse data types, accounting for inter-individual variability, and capturing temporal dynamics are crucial aspects that need to be addressed to ensure the robustness and accuracy of computational models in system vaccinology. To address this, we measure the systems’ response to Tdap booster vaccination over 4 years by creating four independent datasets with different cohorts for which computational models are created and tested (Table 1). We established the Computational Models of Immunity - Pertussis Boost (CMI-PB) resource to develop and test computational models that predict the outcome of Tdap booster vaccination that is designed to be used by the broader community. Here, we report on the outcome of the first challenge: an “internal dry run” where all teams involved in making predictions were part of the grant. We report on the challenges encountered for data sharing, formulating prediction questions, and the interpretation of the results from different prediction models, including the determination of which factors contributed to such predictions. These results will inform the design of the next prediction contest, which will open to community participation in August 2024.

**Table 1:** Past and future CMI-PB annual prediction challenges

	Annual prediction challenge title	Contestants	Number of subjects		Current status
			Training dataset	Test dataset	
1	First challenge: Internal dry run	CMI-PB consortium	60 (28 aP + 32 wP)	36 (19 aP + 17 wP)	concluded in May 2022
2	Second challenge: Invited challenge	invited contestants	96 (47 aP + 49 wP)	23 (13 aP + 10 wP)	concluded in January 2024
3	Third challenge: Open Challenge	public	119 (60 aP + 59 wP)	54^a^ (27 aP + 27 wP)	will be announced in August 2024

Our commitment involves conducting three annual challenges. The first challenge was completed in May 2022 with participation from the CMI-PB consortium. The second challenge concluded in January 2024 and featured the CMI-PB consortium along with a limited number of invited contestants from outside the consortium. We will involve members of the public in the third challenge. The first challenge included training data from a previously published study^7^ and newly generated test data. Similarly, we will use both the training and test data from previous challenges as the training data for future challenges and generate new data for testing purposes.

a Goal

## Results

This section covers two components: first, we describe the experience in setting up and running the internal prediction contest. Second, we describe specific models that were developed and discuss their performance on the prediction tasks.

### Running the prediction contest

#### Providing access to experimental data in a uniform fashion

**Figure 1:**
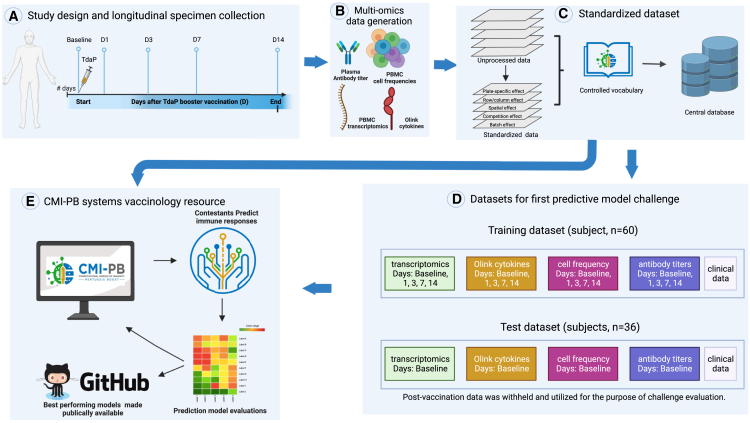
Outline for establishing the CMI-PB resource (A) Recruitment of human subjects and longitudinal specimen collection. (B) Generation of multi-omics data to obtain a comprehensive understanding of the collected specimens. (C) Implementation of a data standardization approach to ensure consistency and comparability of the generated data. (D) The resulting dataset is provided in training and test formats to enable contestants to develop their predictive models. (E) The CMI-PB resource website serves as a platform for hosting an annual prediction challenge, offering data visualization tools for generated data, and providing access to teaching materials and datasets.

Our experimental study is designed for a systems-level understanding of the immune responses induced by Tdap booster vaccination and closely mimics the design of previous studies from our group.^7^ Briefly, individuals primed with aP or wP in infancy were boosted with Tdap and blood was collected pre-booster and post booster at days 1, 3, 7, and 14 (Figure 1A). Multiple assays, including (1) gene expression analysis (RNA sequencing [RNA-seq]) of bulk peripheral blood mononuclear cells (PBMCs), (2) plasma cytokine concentration analysis, and (3) cell frequency analysis of PBMC subsets were performed before and after booster vaccination until day 14. In addition, (4) plasma antibodies against Tdap were measured at all time points. We do not include T cell response assay data in the current challenge but plan to incorporate T cell data in future public CMI-PB challenge. Our overall goal is to make data from these studies available for analysis and utilize it to build computational models that predict the vaccination outcomes of newly tested individuals. For the first CMI-PB challenge, we collected data from a total of 60 subjects (28 aP + 32 wP; Table 1), which can be used as a training dataset to develop predictive models. Additionally, we obtained data from a separate group of 36 newly tested subjects (19 aP + 17 wP), which can be utilized as test data for running predictions.

To integrate experimental data generated at different time points into the centralized CMI-PB database, we created unique subject and sample (specimen) identifiers and provided consistent nomenclatures for the different readouts between training and test datasets. The data collected post vaccination from the test dataset were withheld and utilized for the purpose of challenge evaluation. We used a relational database management system with tables corresponding to entity categories, including subject and specimen information, experimental data, and ontology tables (database schema is provided in Figure S1). We established different access modalities, including an application programming interface (API; https://www.cmi-pb.org/docs/api/) and bulk file downloads, and shared these different access modalities with our internal userbase of contestants.

**Figure 2:**
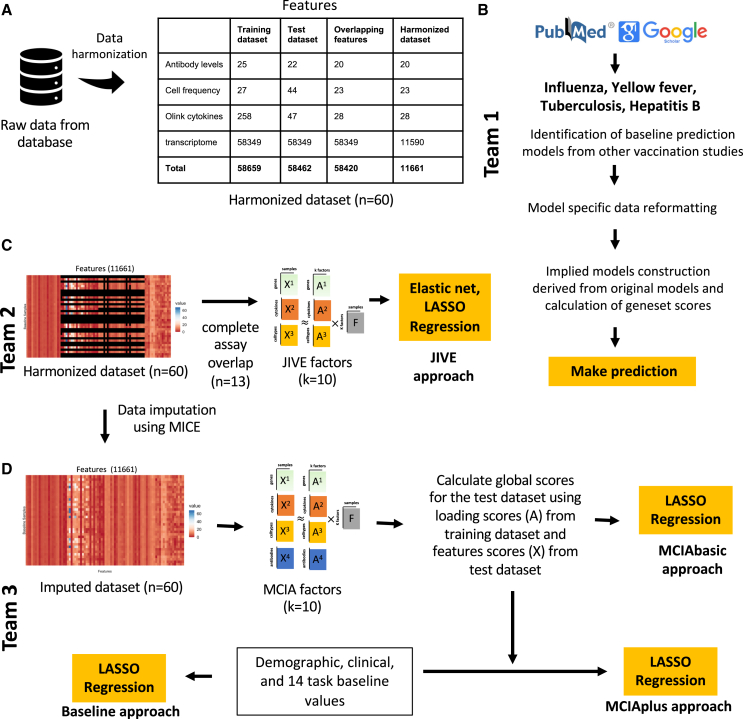
Data processing, computable matrices, and prediction model generation (A) Generation of a harmonized dataset involved identifying shared features between the training and test datasets and filtering out low-information features. Literature-based models (team 1) used raw data from the database and applied data-formatting methods specified by existing models. JIVE and MCIA approaches (teams 2 and 3) utilized harmonized datasets for constructing their models. (B) Flowchart illustrates the steps involved in identifying baseline prediction models from the literature, creating a derived model based on the original models' specifications, and performing predictions as described by the authors. (C) The JIVE approach involved creating a subset of the harmonized dataset by including only subjects with data for all four assays. The JIVE algorithm was then applied to calculate 10 factors, which were subsequently used for making predictions. JIVE employed five different regression models for prediction purposes. (D) MCIA approach applied MICE imputation on the harmonized dataset and used these data for model construction. MCIA method was applied to the training dataset to construct 10 factors. Then, these 10 factors and feature scores from the test dataset were utilized to construct global scores for the test dataset. Lasso regression was applied to make predictions. MCIAplus model was constructed by including additional features (demographic, clinical features, and 14 task values) as factor scores, and it also utilized lasso regression to make predictions. The MCIA approach utilized MICE imputation on the harmonized dataset for model construction. The MCIA method employed the imputed training dataset to construct 10 factors. These 10 factors, along with feature scores from the test dataset, were used to construct global scores for the test dataset. Lasso regression was applied to make predictions. Additionally, the MCIAplus model incorporated additional features such as demographic, clinical features, and 14 task values as factor scores. Finally, lasso regression was employed for making predictions.

The total feature count for the training dataset was 58,659, whereas the feature count for the test dataset was 58,462 (Figure 2A). These large numbers of features were primarily derived from the PBMC gene expression assay dataset, which has 58,343 features with complete feature overlap between the training and test datasets. PBMC frequency assay has 27 features in training and 44 features in the test dataset with 23 overlapping features. Plasma cytokine concentrations assay has 258 features in the training dataset and 47 features in the test dataset with 28 overlapping features. Plasma antibody titers assay has 25 features in training and 22 features in the test dataset with 20 overlapping features.

#### Formulating the prediction tasks

We formulated multiple prediction tasks in order to quantitatively compare different approaches to model immune responses to Tdap booster vaccination. For each prediction task, pre-booster data (except from aP vs. wP status) of each subject were used to predict post-vaccination variables and rank individuals subsequently. We selected biological readouts known to be changed by booster vaccination under the premise that they are likely to capture meaningful heterogeneity across study participants based on our previous work.^7^ For instance, we have shown that the percentage of monocytes was significantly elevated on day 1 post booster vaccination compared to baseline (i.e., before booster vaccination), highlighting the role of monocytes in Tdap vaccine response.^7^ We created a first task in which the overall frequency of monocytes among PBMCs on day 1 post booster vaccination has to be predicted. Similarly, we have shown that plasma immunoglobulin (Ig) G1–4 levels significantly increased at day 7 post booster vaccination compared to baseline.^7^ The second task consists of predicting plasma IgG levels against the pertussis toxin (PT) on day 14 post booster vaccination. The third task is based on our previous finding that a subset of aP-primed individuals showed an increased expression of proinflammatory genes, including *CCL3*, on day 3 post booster vaccination.^7^ This task consists of predicting the gene expression of *CCL3* on day 3 post booster vaccination. Overall, the first challenge comprised 14 prediction tasks that we describe in Table S1, including 13 prediction tasks of readouts identified from previous work and a “sanity-check” task to predict the expression of the sex-specific *XIST* gene post booster vaccination per individual.^35^

#### Choosing a metric to evaluate prediction performance

We set out to choose a metric to evaluate how different prediction methods performed. Specifically, we wanted to have three considerations: (1) we needed a metric that would produce a single numeric value as an output. This would allow us to compare and rank the performance of the prediction methods effectively. (2) The chosen metric needed to be non-parametric because the different experimental assays utilized in the study produce analyte measurement outputs with non-normal distributions. (3) We wanted to avoid incorporating arbitrary cutoffs or thresholds that could introduce subjectivity or bias into the assessment process. Based on these considerations, we chose the Spearman rank correlation coefficient as our primary metric. The prediction tasks in our first challenge thus constituted predicting the rank of individuals in specific immune response readouts from high to low after *B. pertussis* booster vaccination based on their pre-vaccination status.

#### Feedback from participants prior to data submission

We shared the prediction tasks, metrics, and data access instructions with our internal contest participants in order to test our anticipated approach. Two main points of feedback were made prior to receiving prediction results: (1) all users preferred using the bulk file downloads over utilizing the custom API we had created. Upon questioning, most preferred to work with data hands-on rather than having to learn a new interface. Given that creating reliable APIs is resource intensive, this was identified as an area we wanted to down-prioritize going forward. (2) When inspecting the antibody titer data across years, contestants noticed significant variation in the averages of the baseline values for donors (subjects) between the test and training datasets. Those variations were due to a switch in the site where the assays were performed. We thus standardized the antibody data in each year by applying the baseline median as a normalization factor (https://github.com/CMI-PB/2021-Ab-titer-data-normalisation; Figures S2 and S3), and provided both the raw data and normalized data to the contestants.

#### Gathering and evaluating prediction results

**Figure 3:**
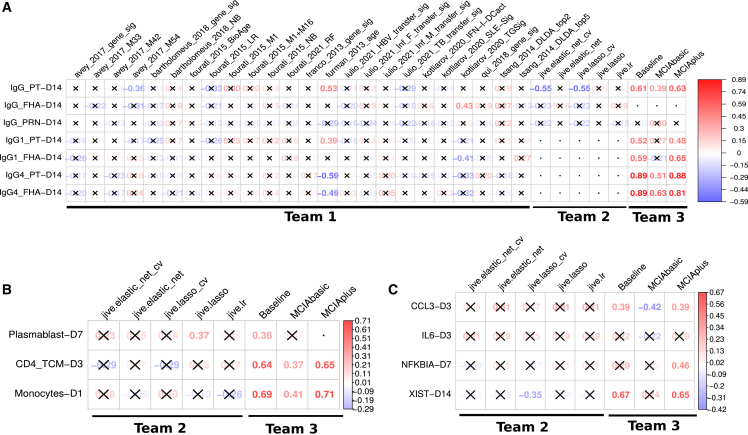
Evaluation of the prediction models submitted for the first CMI-PB challenge Model evaluation was performed using Spearman’s rank correlation coefficient between predicted ranks by a contestant and actual rank for each of (A) antibody titers, (B) immune cell frequencies, and (C) transcriptomics tasks. The number denotes Spearman rank correlation coefficient, while crosses represent any correlations that are not significant using p ≥ 0.05. The baseline and MCIAplus models outperformed other models for most tasks.

A total of 34 computational models were developed by three independent teams in accordance with the theme of the challenge. Each team worked separately on their own set of models. The first team focused on identifying and constructing baseline prediction models based on the systems vaccinology literature (Figure 2B). The second and third teams, on the other hand, focused on constructing prediction models derived from multi-omics dimension-reduction techniques (Figures 2C and 2D). We established a deadline of 3 months for each team to submit their models, and, subsequently, the corresponding predictions were received for evaluation. A complete submission file contained 14 columns, one column per prediction task. We found that most prediction models focused on a subset of tasks. Furthermore, we found that, in some cases, predictions for individual donors were omitted. In those cases, we used the median rank calculated from the ranked list submitted by the contestant to fill in missing ranks. An overview of the prediction results is summarized in Figure 3.

### Model development and evaluation

#### Establishing baseline prediction models from the systems vaccinology literature

With the first team, we set out to identify existing models developed within the systems vaccinology field that aim to predict vaccination outcomes. With systematic keyword queries using PubMed and Google Scholar and following citations, we identified 40 studies of potential interest. A detailed review of these papers identified 10 studies with 24 models that are suitable for our purpose as they (1) used pre-vaccination measurements that we have available in our CMI-PB study, and (2) established biological differences in vaccine responses that we could transfer to our study, either by predicting antibody titers levels or classifying subjects into high or low vaccine-induced antibody responders.^28^^,^^29^^,^^31^^,^^36^^,^^37^^,^^38^^,^^39^^,^^40^^,^^41^^,^^42^ None of these models were developed for *B. pertussis* but rather they cover a wide range of vaccines, including those against influenza, hepatitis B, and the yellow fever virus. They employed a variety of methodologies, including classification-based (diagonal linear discriminant analysis, logistic regression, naive Bayes, random forest), regression-based (elastic net), and other approaches (gene signature and module scores). A summary of the literature review is depicted in Figures 2B and S4 for the 24 prediction methods that were implemented (Table S2). For each literature model, we adapted the output scores to our prediction tasks, as described in the STAR Methods. It has to be emphasized that these models were repurposed for our specific prediction tasks, and our work was not an evaluation of their performance in the areas for which they were intended. Rather, evaluating these adapted models sets a baseline of prediction performance and determines whether universal vaccine response predictors are readily available.

#### Establishing a harmonized dataset to train ML models

Many of the features evaluated by our assays have low-information content, specifically the transcriptomic assay, meaning that they have low analyte levels or analytes absent across specimens. Incorporating less informative features introduces various challenges in data analysis. Low analyte levels could be difficult to distinguish from background noise, missing data could skew statistical analyses, and these features tend to make it more challenging to identify a robust and accurate prediction model. To address these issues, we applied feature filtering on each assay in the training dataset, which is a widely adopted data pre-processing strategy.^7^ For gene expression, we filtered zero variance and mitochondrial genes and removed lowly expressed genes (genes with transcript per million [TPM] <1 in at least 30% of specimens). Similarly, we filtered features with zero variance from cytokine concentrations, cell frequency, and antibody assays. Subsequently, we removed features not measured for the test dataset and retained only those that overlapped between the training and test datasets. As a result, we were left with a total of 11,661 features in the harmonized dataset out of the original 58,420 overlapping features between training and test dataset (Figure 2A).

Multi-omics data typically have many thousands of features and direct model training from such data runs the risk of overfitting, where the model learns the noise in the training data rather than the underlying pattern. For this reason, feature selection techniques and/or domain knowledge are commonly employed to identify and focus on the most informative features, effectively reducing the problem’s dimensionality. We have developed two ML approaches based on the integration of multi-omics data. The harmonized datasets were utilized for training these ML approaches, as described below.

#### Establishing purpose-built models using JIVE

With the second team, we set out to build prediction models using the available CMI-PB training data. Given that this included data from different modalities, we wanted to utilize approaches that could leverage the CMI-PB dataset in an integrative fashion. We thus applied joint dimensionality reduction methods that discover patterns within a single modality and across modalities to reduce the number of dimensions. In particular, we applied the joint and individual variation explained (JIVE) method to reduce the dimensionality of our datasets before applying regression-based models to make predictions.^43^^,^^44^ JIVE decomposes a multi-source dataset into three terms: a low-rank approximation capturing joint variation across sources, low-rank approximations for structured variation individual to each source, and residual noise.^44^ This decomposition can be considered a generalization of principal-component analysis (PCA) for multi-source data.^44^ For JIVE, harmonized datasets for transcriptomics, cell frequency, and cytokines concentrations were first intersected on subjects, which resulted in 13 individuals with complete data, and, finally, the decomposition was applied, generating 10 factors per omics (Figure 2C). These factors were then used as input for five different regression-based methods to turn the JIVE results into predictive models for each specific task. These regression methods included linear regression, lasso, and elastic net with default parameters and two more variants of lasso and elastic net that involved an automatic hyperparameter search via cross-validation (CV; see Figure 2C).

#### Establishing purpose-built models using multiple co-inertia analysis

The third team worked on three different approaches to build prediction models (Figure 2D). The first approach (baseline approach) utilized clinical features (age, infancy vaccination, biological sex) and baseline task values as predictors of individual tasks. The second approach (the MCIAbasic) utilized 10 multi-omics factors constructed using multiple co-inertia analysis (MCIA) as predictors of individual tasks. Prior to implementing MCIA, the harmonized datasets were further processed to impute missing data in the baseline training set using the multiple imputation by chained equations (MICE) algorithm (Figure 2).^45^ The objective function in MCIA maximizes the covariance between each individual omic and a global data matrix consisting of the concatenated omic data blocks.^46^^,^^47^ Finally, the third approach (MCIAplus) combined the first two approaches and utilized clinical features, baseline task values, the baseline approach, and 10 MCIA factors identified through the MCIAbasic approach as predictors of individual tasks. Further, for all three approaches, we built a general linear model with lasso regularization for each task. We used the feature scores as input data and the prediction task values as response variables, generating separate predictive models for each task.

#### Comparing model prediction performance

In total, 32 different model predictions were submitted across the tasks, including 24 models identified from the literature to address antibody-related tasks, as well as eight models derived from multi-omics dimension reduction techniques, such as JIVE and MCIA. A heatmap visualization of Spearman’s correlations for tasks versus models is presented in Figure 3. At least one of the prediction models showed significant correlations for 10 out of 12 prediction tasks, whereas no model showed significant correlations for the remaining two tasks.

The 24 literature-based models were specifically designed to address antibody-related tasks and gave insignificant correlations for 22 out of 24 models. Exceptions were two models (furman_2013_age and kotliarov_2020_TGSig), showing significant correlations for six out of the seven antibody-related tasks by at least one model. The most successful model (furman_2013_age) was derived from a previous study by Furman et al.,^38^ where chronological age of an individual was used as the sole predictor for antibody response levels to influenza vaccination. Signature-based analyses, such as pathway and clustering analysis, are effective in capturing patterns within omics datasets that have a large number of variables, including transcriptomic data. However, when it comes to age-based signatures, a primary limitation arises from datasets where the range of ages of individuals is very limited. In our specific case, both our training and test cohorts have a median age of 23 years, with a range of 18–51 and 19–47 years old, respectively. Despite this limitation, the furman_2013_age model successfully predicted tasks associated with PT-specific IgG and its subtypes, IgG1 and IgG4 (Figure 2). Many models described that biological age, measured through an individual’s physiological state and overall health, is more accurate than chronological age for predicting the onset of disease and death.^37^^,^^48^ We examined a derived model incorporating biological age from Fourati et al.^37^ to compare whether this model had similar performance to chronological age, which did not demonstrate significant correlations. These results suggest that chronological age has a strong predictive potential to be universally utilized as a biomarker to predict antibody responses against different pathogens in addition to influenza and *B. pertussis*. The second-best-performing implied model (kotliarov_2020_TGSig) employed signature analysis using blood transcription modules (BTMs) and established sets of transcriptional modules designed to describe the changes in gene expression in blood in response to different vaccines.^49^ In the case of the kotliarov_2020_TGSig model, a specific BTM comprising B cell gene signatures was utilized as the predictor of antibody response levels to influenza vaccination in the original studies, and it successfully predicted tasks related to filamentous hemagglutinin (FHA)-specific IgG and IgG1 responses. Overall, the majority of literature-based implied models exhibited insignificant performance, while the simplest model that solely relied on chronological age demonstrated promising results.

JIVE-based submissions attempted 10 tasks, excluding the four antibody-related tasks that had missing samples within the harmonized dataset. Diving into the cell-frequency tasks, we saw a modest performance for predicting plasmablast levels on day 7, and, surprisingly, the simple linear regression performed best. However, for other cell-frequency tasks, there was no clear pattern of model performance. Within gene-expression tasks, JIVE-based models performed best when predicting *CCL3* levels on day 3 and, once again, models without hyperparameter tuning performed the best. Hyperparameter tuning is a procedure that requires a set of candidate values for each hyperparameter; for lasso and elastic net, this means optimizing alpha and/or L1 ratio. In ideal situations, models derived from hyperparameter tuning should perform the best; however, given our low number of samples, this process may become unstable and lead to overfitting. Turning to *IL6* at day 3, all JIVE-based models performed modestly, which suggests that this task may be harder than others. As for *NFKBIA* on day 7, predictions were poor for all JIVE-based models. The poor performance of JIVE-based models on some predictive tasks may be due to the limited number of subjects used (n = 13). A recent benchmark paper showed that JIVE performs reasonably well with a dataset of approximately 170 samples.^50^ Another issue may be that the latent factors learned by JIVE are not necessarily capturing correlates of predictive tasks framed in this challenge without inclusion of any clinical information and explicit use of baseline values for each task. Going forward, we will continue utilizing JIVE-based models but will try to improve them by utilizing more samples, more latent factors, and clinical information for the future iterations of this challenge.

The baseline, MCIAbasic, and MCIAplus approaches were the only methods that submitted predictions for all 14 tasks. These three approaches outperformed other teams' approaches. Specifically, both the MCIAplus and baseline approaches demonstrated significant correlations for 10 out of the 14 tasks, as illustrated in Figure 3. On the other hand, the MCIAbasic approach exhibited significant positive correlations for five out of the 14 tasks. When examining the antibody tasks, both MCIAplus and the baseline approaches showed robust performance, ranking first in five out of the seven tasks. The baseline approach showed significant correlations for all three cell-frequency tasks, whereas MCIAplus has similar performance to the baseline model for two tasks except for predicting plasmablast on day 7. The MCIAplus model showed significant correlations for three out of four gene-expression tasks, while the baseline model showed significant correlations for two out of four tasks. The MCIAbasic approach worked very well with three antibody levels and two cell-frequency tasks; however, it performed poorly for all four gene-expression tasks. When examining what factors led to improved performance of the baseline and MCIAplus approach as compared to the MCIAbasic approach, it was straightforward to deduce that clinical information and the baseline values of the prediction tasks were strong contributors in predicting most tasks. However, there was one notable exception observed in the analysis. Specifically, when considering the task related to *NFKBIA* on day 7, the MCIAplus approach exhibited a significant correlation, outperforming both the baseline and MCIAbasic models. This improvement in performance was attributed to a combination of MCIA factors and baseline features, highlighting their collective contribution to the predictive capabilities of the MCIAplus approach in this particular task. We noted that, while most contributing features were shared between the baseline and MCIAplus approaches for most tasks, there were certain instances where the MCIA factors exhibited a greater contribution. For example, in the prediction of IgG responses on day 14, common features were baseline levels of IgG and IgG1 responses against PT; however, apart from these two features, two MCIA factors were significant contributors to the MCIAplus model, whereas baseline levels of IgG1 responses against FHA antigen were a significant contributor in the case baseline model. Overall, it is worth noting that clinical information and baseline values of known immune signatures significantly affected the prediction performance of underlying models.

## Discussion

Here, we report on the first rigorous evaluation of multi-omics prediction tools on vaccine immune responses. This inaugural dry run constitutes an important step for the development and refinement of our future community prediction contest. Furthermore, all source code for the imputation, models, and assessment metrics is publicly available as part of our CMI-PB GitHub repository (https://github.com/CMI-PB). This will serve as an important resource and benchmark for future contestants.

Major lessons learned from our inaugural prediction contest include the importance of providing contestants with both original (raw) data and standardized computable matrices. Through this approach, we can simplify the process of data access and help avoid contestants having to standardize their model inputs independently. Also highlighted was the importance of testing the compatibility across all data sources before announcing the challenge, as we realized that additional normalization was required for the antibody titer data. Critically, we also learned that clinical variables, such as age, can play a role in making successful predictions, and thus we have included all collected clinical information, including health-span-related characteristics such as chronic diseases and immune exposures, in all future challenge datasets. We are expanding the CMI-PB challenge to over 30 invited contestants to validate our approach for a second time before opening the next round to the public. This second CMI-PB challenge has been designed to address some of the shortcomings identified during the first challenge. We expect to make additional adjustments informed by the second challenge to help ensure success in the initial public challenge. This iterative process aims to provide contestants with a rich user experience, allowing for smoother data access and a much less tedious prediction submission process.

The major goal of the first challenge was to develop and refine a pipeline that can access methods for predicting the immune response to Tdap booster vaccination. The pipeline developed to run the first challenge provided a benchmark for models developed in future contests and code to evaluate the performance and significance of the results. In order to identify biomarkers that are generally important for a successful vaccination response, a large number of samples is needed, divided across multiple cohorts. In the coming years, additional datasets will become available within the CMI-PB resource. This will undoubtedly assist the development and tailoring of models specifically aimed at predicting the immune response outcomes of the Tdap vaccination. With several Tdap vaccination cohorts, it should be possible to determine the components of the immune response that are consistently important for a good vaccine response.

The presented results based on literature models demonstrated that the majority of vaccine prediction methods found in the literature are inadequate in capturing the fundamental immunological features required for effective vaccination against *B. pertussis*. Several plausible explanations exist for the lack of generalizability and insufficient capture of underlying mechanisms in these prediction methods. One possibility is that vaccinations for distinct pathogens possess fundamentally unique characteristics. Another potential explanation is that these prediction methods may be overfitting the datasets used for their development, which is a well-known problem in ML models that require training data for prediction.^29^ In the present study, the ability of transcriptional, clinical, and cell population-based signatures to predict vaccine responses was independently examined across multiple studies from the literature. The results showed that only occasionally were the immune signatures significant in a study from which they were not derived. This indicates that prediction methods that are developed on a single or a few vaccination studies usually do not generalize well. It is noteworthy that a model based on age inferred from the literature exhibited strong performance in predicting antibody-related tasks. Aging is characterized by a progressive loss of physiological integrity and an increased susceptibility to immunosenescence.^51^ Age has been reported to be an important determinant of vaccine effectiveness in older adults.^52^ Furthermore, we plan to incorporate more clinical factors, including immune exposures, time of vaccination, and health history attributes, into all future contests. This will aid contestants in constructing more refined prediction models.

The presented results, based on the JIVE and MCIA ML approaches, provide valuable insights into the importance of data imputation, model quality check, and the significant impact of incorporating clinical and pre-vaccination signatures on model performance. The baseline approach was the simplest modeling approach among all and attained notable performance for most tasks. This finding aligns with recent demonstrations that integrating prior immunological knowledge serves as an effective approach for reducing model complexity and improving robustness.^53^ Further, it is worth noting that MCIA and JIVE are distinct extensions of PCA, each employing different algorithms to decompose information extracted from multi-omics datasets. It is important to clarify that our intention was not to compare these two models directly but rather to share our learnings from the two separate prediction approaches. With the JIVE approach, we opted to use complete assay information for model development with minimal data pre-processing. However, this approach yielded limited success, likely due to the limited sample size after requiring complete data for each subject, except for moderate performance in predicting two specific tasks. We are keen to refine further the JIVE approach in alignment with the MCIAplus approach in future challenges. Similarly, MCIAbasic model implementation closely resembled JIVE, except for the utilization of imputed data. With the imputed data, this model achieved significant success as compared to JIVE. Unsupervised approaches such as MCIA hold a lot of potential in uncovering hidden patterns and relationships within complex immune profiles. Further, the MCIAplus approach performed significantly well in predicting most tasks where we integrated modeling, immunological insights, and clinical knowledge together. We intend to reapply this model for future challenges and look forward to improving the MCIAplus approach with pre-vaccination immune signatures available within existing studies, such as utilizing BloodGen3 modules to identify pertussis booster pre-vaccination signatures.^49^ Overall, our ML approaches pointed out that, beyond age, the inclusion of baseline responses also was a key determining factor to get predictions right. In the next challenge cycle, we expect every contestant to recognize this and integrate it into their approach for improved results.

With the first challenge, we focused on a limited set of prediction approaches, including the existing baseline models from the literature and two ML-based models. There are plenty of other approaches that have been utilized to elucidate the kinetics of vaccine-induced immune responses and the durability of vaccine effects. For instance, network-based and longitudinal modeling approaches utilized dynamic patterns and temporal relationships between omics to predict vaccine responses.^54^^,^^55^ Our cohort size will be growing with the recruitment of study subjects for each future challenge, and we believe this would help prediction models perform better as larger datasets provide a richer and more diverse pool of information, allowing models to capture more complex patterns and relationships, leading to improved predictive performance and generalization capabilities. This expanded data volume will also help mitigate issues such as overfitting and enhance the models' robustness and reliability. In addition, we are considering to include T cell assay data, which will become available for future challenges. In the first challenge, our emphasis was primarily on the execution and evaluation of the contest pipeline, rather than delving into the biological rationale underlying the top-performing models. We intend to study top-performing models from upcoming contests closely, and we believe this will greatly aid in comprehending the influence of various factors that contributed to the accurate prediction of existing Tdap booster vaccination signatures. In the first challenge, we incorporated a total of 14 tasks, out of which contestants successfully generated significant predictions for 12 tasks. However, two tasks pertaining to IgG response to pertactin (PRN) antigen on day 14 and *IL6* expression on day 3 did not yield significant predictions. We will continue to evaluate which prediction tasks are the most meaningful; what are the right data to evaluate them; and how questions should be asked, such as asking for an absolute ranking of responses or a fold change compared to baseline.

We are committed to performing comparable experiments on a yearly basis that can be used to build a large set of consistent experimental data. The CMI-PB resource (1) provides access to systems vaccinology data from prior experiments by our group and others relevant to Tdap booster vaccination, (2) explains the nature of the experiments performed and the data generated and how to interpret them (which can be a hurdle for more computationally oriented scientists), and (3) invites visitors to participate in the prediction challenge that asks to utilize baseline data from individuals prior to vaccination in order to predict how they rank in different vaccine response measurements. We believe that the open access to data and the ability to compare model performances will increase the quality and acceptance of computational models in systems vaccinology.

We believe that this collaborative and innovative approach will create a hub for immunologists to push for novel models of immunity against Tdap boost. We expect the resultant models will also be relevant for other vaccinology studies. Contestants from the research community that are interested in participating are encouraged to contact us via cmi-pb-contest@lji.org and check the website (www.cmi-pb.org) for the upcoming contest information.

### Limitations of the study

The primary limitation of our study is that we may have missed published models that perform better than what we have reported. Given the open nature of our performance evaluation, we welcome all developers of published models to submit their predictions for this study or the planned upcoming prediction contests.

## STAR★Methods

### Key resources table

**Table:** 

REAGENT or RESOURCE	SOURCE	IDENTIFIER
**Antibodies**		

IgG mouse anti-human (PE, clone JDC-10)	SouthernBiotech	9040–09; RRID:AB_2796601
IgG1 mouse anti-human (PE, clone HP6001)	SouthernBiotech	9054–09; RRID:AB_2796628
IgG2 mouse anti-human (PE, clone HP6025)	SouthernBiotech	9070–09; RRID:AB_2796639
IgG3 mouse anti-human (PE, clone HP6050)	SouthernBiotech	9210–09; RRID:AB_2796701
IgG4 mouse anti-human (PE, clone HP6025)	SouthernBiotech	9200–09; RRID:AB_2796693
IgE mouse anti-human (PE, BE5)	Thermo Fisher	MA1-10375; RRID:AB_2536764
Anti-Human CD45 (CyTOF, 89Y, clone HI30)	Fluidigm	3089003B; RRID: AB_2661851
Anti-Human CD3 (CyTOF, 115In, clone UCHT1)	Biolegend	300443; RRID:AB_2562808
Anti-Human CD19 (CyTOF, 142ND, clone HIB19)	Fluidigm	3142001B; RRID: AB_2651155
Anti-Human CD38 (CyTOF, 144ND, clone HIT2)	Fluidigm	3144014B; RRID: AB_2687640
Anti-Human CD4 (CyTOF, 145ND, clone RPA-T4)	Fluidigm	3145001B; RRID: AB_2661789
Anti-Human CD20 (CyTOF, clone 2H7)	Biolegend	302302; RRID:AB_314250
Anti-Human CD123 (CyTOF, 151Eu, clone 6H6)	Fluidigm	3151001B; RRID: AB_2661794
Anti-Human CD45RA (CyTOF, 155Gd, clone HI100)	Fluidigm	3155011B; RRID: AB_2810246
Anti-Human CD1c (CyTOF, 160Gd, clone L161)	Biolegend	331502;RRID:AB_1088996
Anti-Human CD33 (CyTOF, 163Dy, clone WM53)	Fluidigm	3163023B; RRID: AB_2687857
Anti-Human CCR7 (CyTOF, 167Er, clone G043H7)	Fluidigm	3167009A; RRID: AB_2858236
Anti-Human CD25 (CyTOF, 169Tm, clone M-A251)	Fluidigm	3169003B; RRID: AB_2661806
Anti-Human CD8a (CyTOF, 172Yb, clone RPA-T8)	Biolegend	301002; RRID:AB_314120
Anti-Human CD14 (CyTOF, 173Yb, clone 61D3)	Thermo Fisher	14-0149-82; RRID:AB_467129
Anti-Human HLA-DR (CyTOF, 174Yb, clone L243)	Fluidigm	3174001B; RRID: AB_2665397
Anti-Human CD56 (CyTOF, 176Yb, clone CMSSB)	Fluidigm	3176003B; RRID: AB_2756430
Anti-Human CD16 (CyTOF, 209Bi, clone 3G8)	Fluidigm	3209002B; RRID: AB_2756431

**Biological samples**		

PBMC and Plasma	LJI Clinical Core	https://www.lji.org/research/research-services/clinical-studies/

**Chemicals, peptides, and recombinant proteins**		

Pertussis Toxin (PT)	List Biologicals	180
Tetanus Toxoid (TT)	List Biologicals	191A
Pertactin (PRN)	List Biologicals	187
Fimbriae 2/3 (Fim2/3)	List Biologicals	186
Diphtheria Toxoid (DT)	List Biologicals	151
Filamentous Hemagglutinin (FHA)	Sigma	F5551-50UG
Ovalbumin (OVA)	Sigma	vac-stova
MagPlex-C Microspheres	Luminex	MC10012-01 MC10019-01 MC10025-01 MC10034-01 MC10037-01 MC10042-01 MC10048-01
Zeba TM Spin Desalting Columns 7K MWCO	Thermo Scientific	89882
Ficoll-Paque PLUS	GE	17144003
RPMI 1640 medium	Omega Scientific	RP-21
DMSO	Sigma	D8418
QIAzol Lysis Reagent	Qiagen	79306
Human Pertussis antiserum	NIBSC	06/140
Cell-ID Intercalator-Ir	Fluidigm	NC1038184

**Critical commercial assays**		

Target 96 Immuno-oncology panel	Olink	https://olink.com/products-services/target/immune-response-panel/
Target 96 Immune response panel	Olink	https://olink.com/products-services/target/immune-response-panel/
Target 96 Metabolism panel	Olink	https://olink.com/products-services/target/biological-process/
xMAP® Antibody Coupling Kit	Luminex	40–50016
MAGPIX Calibration Kit	Luminex	MPX-CAL K25
MAGPIX Performance Verification Kit	Luminex	MPX-PVER-K25
miRNeasy Mini Kit	Qiagen	217084
TruSeq Stranded mRNA Library Prep Kit	Illumina	20020595

**Deposited data**		

bulkRNAseq, PBMC subset frequencies, plasma antibody measurements, plasma cytokine measurements.	This paper	https://www.cmi-pb.org/data; https://doi.org/10.5281/zenodo.10789473

**Software and algorithms**		

R (V4.0.1 - V4.3.1)	R Core Team (2020)	https://www.r-project.org/
FlowJo software version 10.7.0	BD Biosciences	https://www.flowjo.com/
Python (v3.7+)	Python community	https://www.python.org/
PostgreSQL (V4)	PostgreSQL Team	https://www.postgresql.org/
JIVE	Lock et al.^44^	https://cran.r-project.org/web/packages/r.jive/index.html
Multiple co-inertia analysis (MCIA)	Meng et al.^46^	https://bioconductor.org/packages/release/bioc/html/omicade4.html
GLMNET		https://cran.r-project.org/web/packages/glmnet/index.html
STAR (v2.6.1)	Dobin et al.^56^	https://github.com/alexdobin/STAR
PRINSEQ Lite (v0.20.3)	Schmieder & Edwards^57^	https://github.com/uwb-linux/prinseq
SAMtools	Li et al.^58^	https://github.com/samtools/samtools
featureCounts (v1.6.5)	Liao et al.^59^	https://subread.sourceforge.net/
Antibody titer data normalization	This paper	https://doi.org/10.5281/zenodo.10642095
Generation computable matrices and MCIA model	This paper	https://doi.org/10.5281/zenodo.10642081
24 models derived using the literature-based survey	This paper	https://doi.org/10.5281/zenodo.10642079
JIVE models	This paper	https://doi.org/10.5281/zenodo.10642104

### Resource availability

#### Lead contact

Further information and requests for resources and reagents should be directed to and will be fulfilled by Lead Contact, Bjoern Peters (bpeters@lji.org).

#### Materials availability

This study did not generate new unique reagents.

#### Data and code availability

• The training and test datasets used for the first challenge can be accessible through a Zenodo repository at https://doi.org/10.5281/zenodo.10789473. The repository includes detailed information on the datasets, challenge tasks, submission format, submission files and evaluation code, descriptions, and access to the necessary data files that contestants used to develop their predictive models and make predictions.
• The codebase for normalizing antibody titer data is available at Zenodo (https://zenodo.org/records/10642152), while the code for standardizing data and generating computable matrices is available at Zenodo (https://doi.org/10.5281/zenodo.10642081). The codes for all models submitted for the first CMI-PB challenge are available, including those identified from the literature. All 24 models derived using the literature-based survey are available at Zenodo (https://zenodo.org/records/10642081). The codebase for the JIVE models is available at Zenodo (https://zenodo.org/records/10642104) and the codebase for the MCIA-based models can be found at Zenodo (https://zenodo.org/records/10642081).
• Any additional information required to reanalyze the data reported in this work paper is available from the lead contact upon request.

### Experimental model and study participant details

Human volunteers that were primed with either the aP or wP vaccination during childhood were recruited. The characteristics of all participants are summarized in Table S4. All participants provided written informed consent before donation and were eligible for Tdap (aP) booster vaccination containing tetanus toxoid (TT), diphtheria toxoid (DT), and acellular Pertussis that contains inactivated pertussis toxin (PT) and cell surface proteins of Bordetella pertussis including filamentous hemagglutinin (FHA), fimbriae 2/3 (Fim2/3), pertactin (PRN). Longitudinal blood samples were collected pre-booster vaccination (day 0) and post-booster vaccination after 1, 3, 7, and 14 days. This study was performed with approvals from the IRB at the La Jolla Institute for Immunology, and written informed consent was obtained from all participants before enrollment.

### Method details

#### PBMC and plasma extraction

Whole blood samples (with heparin) were centrifuged at 1850 rpm for 15 min with breaks off. Subsequently, the upper fraction (plasma) was collected and stored at −80°C. PBMCs were isolated by density gradient centrifugation using Ficoll-Paque PLUS (GE). 35 mL of RPMI 1640 medium (RPMI, Omega Scientific) diluted blood was slowly layered on top of 15 mL Ficoll-Paque PLUS. Samples were spinned at 1850 rpm for 25 min with breaks off. Then, PBMC layers were aspirated and two PBMC layers per donor were combined in a new tube together with RPMI. Samples were spinned at 1850 rpm for 10 min with a low break. Cell pellets of the same donors were combined and washed with RPMI and spinned at 1850 rpm for 10 min with breaks off. Finally, PBMCs were counted using trypan blue and a hemocytometer and, after another spin, resuspended in FBS (Gemini) containing 10% DMSO (Sigma-Aldrich) and stored in Mr. Frosty cell freezing container overnight at −80°C. The next day, samples were stored at liquid nitrogen until further use.

#### Plasma antibody measurements

Pertussis antigen-specific antibody responses were quantified in human plasma by performing an indirect serological assay with xMAP Microspheres (details described in xMAP Cookbook, Luminex 5^th^ edition). Pertussis, Tetanus, and Diphtheria antigens (PT, PRN, Fim2/3, TT, and DT (all from List Biological Laboratories) and FHA (Sigma) and as a negative control Ovalbumin (Sigma) were coupled to uniquely coded beads (xMAP MagPlex Microspheres, Luminex Corporation). PT was inactivated by incubation with 1% formaldehyde (PFA) at 4°C for 1 h. 1% PFA PT and TT were then purified using Zeba spin desalting columns (ThermoFisher). The antigens were coupled with each unique conjugated microsphere using the xMAP Antibody Coupling Kit (Luminex Corporation). Plasma was mixed with a mixture of each conjugated microsphere, and WHO International Standard Human Pertussis antiserum was used as a reference standard (NIBSC, 06/140). Subsequently, the mixtures were washed with 0.05% TWEEN 20 in PBS (Sigma-Aldrich) to exclude non-specific antibodies, and targeted antibodies responses were detected via anti-human IgG-PE, IgG1-PE, IgG2-PE, IgG3-PE, IgG4-PE (all from SouthernBiotech) and human IgE-PE (ThermoFisher). Samples were subsequently measured on an FLEXMAP 3D instrument (Luminex Corporation), and the log(10) of the median fluorescent intensity (MFI) was calculated.

#### PBMC cell frequencies

Cryopreserved PBMC were thawed by incubating cryovials at 37°C for 1 min and stained with the viability marker Cisplatin. Subsequently, PBMCs were incubated with an antibody mixture for 30 min. After washing, PBMCs were fixed in PBS (Thermo Fisher) with 2% PFA (Sigma-Aldrich) overnight at 4°C. The next day, PBMCs were stained with an intracellular antibody mixture after permeabilization using saponin-based Perm Buffer (eBioscience). After washing, cellular DNA was labeled with Cell-ID Intercalator-Ir (Fluidigm) and cell pellets were resuspended in 1:10 EQ Beads (Fluidigm) in 1 mL MiliQ water. Samples were measured using a Helios mass cytometer (Fluidigm). Twenty One different PBMC cell subsets were identified using the unsupervised gating approach DAFi^60^ with the exception of antibody-secreting cells (ASCs), which were manually gated as CD45^+^Live^+^CD14^−^CD3^−^CD19^+^CD20^−^CD38^+^ cells. Gating was performed using FlowJo (BD, version 10.7.0).

#### Plasma cytokine concentrations

Plasma samples were randomly distributed on 96 well plates for quantification. 276 different proteins (immuno-oncology, immune response, and metabolism Olink panels) were quantified by Analysis Lab at Olink Proteomics. Protein quantification involved the Proximity Extension Assay (PEA) technology.^61^ Briefly, the plasma was incubated with oligonucleotides labeled antibodies targeting the proteins of interest. The oligonucleotides of matched oligonucleotides-antibodies-antigen will bind to each other, enabling amplification and thereby quantification by qPCR. Ct values from the qPCR were used to calculate Normalized Protein eXpression (NPX), a relative quantification unit to report protein expression levels in plasma samples.

#### RNA sequencing

Per sample, 6 million PBMCs were lysed using QIAzol Lysis Reagent (Qiagen). Samples were stored at −80°C until RNA extraction. RNA was extracted using the miRNeasy Mini Kit (Qiagen) including DNase treatment according to the manufacturer’s instructions. 500 ng of RNA was used for RNA sequencing (RNAseq) library preparation. Library preparation was performed using the TruSeq Stranded mRNA Library Prep Kit (Illumina). Libraries were sequenced on a HiSeq3000 (Illumina) system.

#### Bioinformatics RNA sequencing

The paired-end reads that passed Illumina filters were further filtered for reads aligning to tRNA, rRNA, adapter sequences, and spike-in controls. The remaining reads were aligned to the GRCh38 reference genome and Gencode v27 annotations using STAR (v2.6.1).^56^ DUST scores were calculated with PRINSEQ Lite (v0.20.3),^57^ and low-complexity reads (DUST >4) were removed from the BAM files. The alignment results were parsed via the SAMtools^58^ to generate SAM files. Read counts to each genomic feature were obtained with the featureCounts (v1.6.5)^59^ using the default options along with a minimum quality cut-off (Phred >10).

#### Computational models development

##### Establishing baseline prediction models from the systems vaccinology literature

For literature models that did not present a quantification of the gene sets, a gene set output score was developed. The first step of the calculation was to separate genes that were up- and down-regulated. Next, for each specimen, i the TPM normalized gene expression counts were summed for the upregulated genes ($Sum_{UP}$) and the downregulated genes ($Sum_{DOWN}$). Then the difference between $Sum_{UP}$ and $Sum_{DOWN}$ was calculated for each specimen:

$$X_{i}=Sum_{UP}\;-\;Sum_{DOWN}$$

The average (Avg) and the standard deviation (Std) of the TPM normalized gene expression was calculated across all specimens as well as the square root of the total number of specimens N. Finally, a standard score (zscore) was calculated for each specimen:

$$zscore_{i}=\left(X_{i\;}-\;Avg\right)/\left(Std/\sqrt{N}\right)$$

If there were only upregulated genes, or if it could not be determined whether the genes in the gene signature were up- or down-regulated, the sum of the genes in the signature was simply used for the calculation of the zscore.

##### Establishing purpose-built models using JIVE

To develop models using JIVE we used harmonized datasets for transcriptome, antibody levels and cytokine levels and located sample values for every variable, in other words, complete datasets which resulted in 13 individuals (Figure 2C). Decomposition with JIVE was then applied by using the r.jive package with *jive(omics, rankJ=10, rankA = rep(10, 3)), method = "given", conv = "default", maxiter = 100, showProgress=FALSE)* which resulted in 10 factors and saved the factor loading values.^62^ In order to make a model from a reduced representation of the omics datasets, each omic was multiplied by its corresponding factor loadings to generate factor scores. During training, these factor scores were used as input into various models and approaches from scikit-learn python package,^63^ specifically, we used basic linear regression (LinearRegression), lasso (Lasso), elastic net (ElasticNet), and lastly, lasso and elastic net with automatic hyperparameter tuning via cross-validation (LassoCV and ElasticNetCV). For methods using automatic hyperparameter tuning, candidate values were not specified, therefore using the internal heuristics, which tests hyperparameter values of various magnitudes. During the prediction steps, the testing data is projected onto the lower dimensional space by multiplying the factor loadings by the omics datasets. These new factor scores were then used as input for predictions and finally ranked before contest submission.

##### Establishing purpose-built models using MCIA

The MICE (Multiple Imputation by Chained Equations) method was employed to replace missing data values in the harmonized dataset (Figure 2D). Specifically, transcriptome data was utilized to impute missing values in other data modalities through the application of MICE. We utlilized MICE imputed data to construct models. We implemented MCIA using the mbpca function in the *mogsa* package.^46^^,^^64^ We generated 10 low-dimension multi-omics factor scores for training datasets. Each multi-omics factor was derived through a linear combination of the original features (e.g., genes or proteins) extracted from the input data. Subsequently, global scores were computed for the test dataset, capturing the overall representation or summary of the data in relation to the underlying factors identified from the training data. This was accomplished by utilizing factor loadings from the training dataset and feature scores from the test dataset. For each task, we constructed a prediction model utilizing a general linear model with lasso regularization using the *glmnet* library.^65^ We used the feature scores as input data and the prediction task values as response variables, generating separate predictive models for each task.

### Quantification and statistical analysis

Statistical analyses are detailed for each specific technique in the specific Methods section or in the figure legends, where each specific comparison is presented. Statistical tests were performed using R (version 4.1, www.r-project.org/) of Spearman correlation coefficient. Details pertaining to significance are also noted in Figure 3 legends, and p < 0.05 defined as statistical significance.

## Supplemental information

Document S1. Figures S1–S4 and Tables S1–S4

Document S2. Article plus supplemental information
