# Supplementary material for: A multi-omics systems vaccinology resource to develop and test computational models of immunity
Source: Cell Rep Methods. 2024 Mar 14;4(3):100731. doi: 10.1016/j.crmeth.2024.100731 (PMC10985234; doi:10.1016/j.crmeth.2024.100731)
Supplement: Document S1. Figures S1–S4 and Tables S1–S4 [file mmc1.pdf]

Cell Reports Methods, Volume *4*

Supplemental information

A multi-omics systems vaccinology resource to develop and test computational models of immunity

Pramod Shinde, Ferran Soldevila, Joaquin Reyna, Minori Aoki, Mikkel Rasmussen, Lisa Willemsen, Mari Kojima, Brendan Ha, Jason A. Greenbaum, James A. Overton, Hector Guzman-Orozco, Somayeh Nili, Shelby Orfield, Jeremy P. Gygi, Ricardo da Silva Antunes, Alessandro Sette, Barry Grant, Lars Rønn Olsen, Anna Konstorum, Leying Guan, Ferhat Ay, Steven H. Kleinstein, and Bjoern Peters

Cell Reports Methods, Volume *4*

Supplemental information

A multi-omics systems vaccinology resource

to develop and test

computational models of immunity

Pramod Shinde, Ferran Soldevila, Joaquin Reyna, Minori Aoki, Mikkel Rasmussen, Lisa Willemsen, Mari Kojima, Brendan Ha, Jason A. Greenbaum, James A. Overton, Hector Guzman-Orozco, Somayeh Nili, Shelby Orfield, Jeremy P. Gygi, Ricardo da Silva Antunes, Alessandro Sette, Barry Grant, Lars Rønn Olsen, Anna Konstorum, Leying Guan, Ferhat Ay, Steven H. Kleinstein, and Bjoern Peters

Supplementary Figures

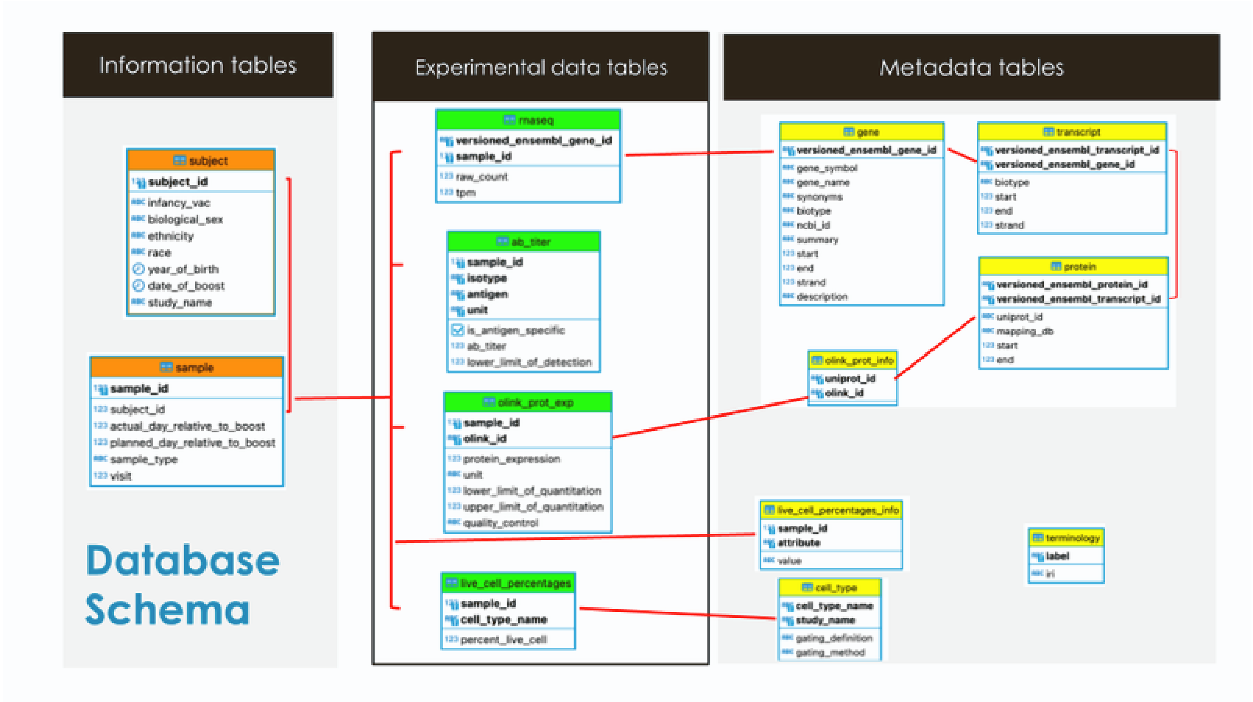

Figure S1: CMI-PB central database schema. The database schema is divided into information, experimental data, and metadata tables. The information tables capture subject and sample information, while the experimental data tables capture experimental data for each omics. In the *rnaseq* table, each row represents an Ensembl gene identifier (versioned_ensembl_gene_id) along with specimen id (specimen_id) and values for measured analytes (raw_count and tpm_count). The *ab_titer* table contains a row for each specimen (specimen_id), with the names and values for measured analytes (isotype, is_antigen_specific, antigen, ab_titer, unit, and lower_limit_of_detection) for the antibody titer experiment. The *olink* table contains a row for each specimen (specimen_id), with the names and values for measured analytes (olink_id and protein_expression). Each row in the *live_cell_percentages* table represents a specimen (specimen_id) along with the names and values for measured analytes (cell_type_name and percent_live_cell). Information on the mapping between olink_id and uniprot_id can be extracted using the *olink_prot_info* table. The metadata tables capture information about ID mapping between CMI-PB data and external databases. The gene, transcript, and protein tables map Ensembl gene, transcript, and protein IDs. These Ensembl IDs are then mapped to UniProt IDs. The *live_cell_percentages_info* and *cell_type* tables provide information about gating information and the experimental technique used to run cell frequency experiments.

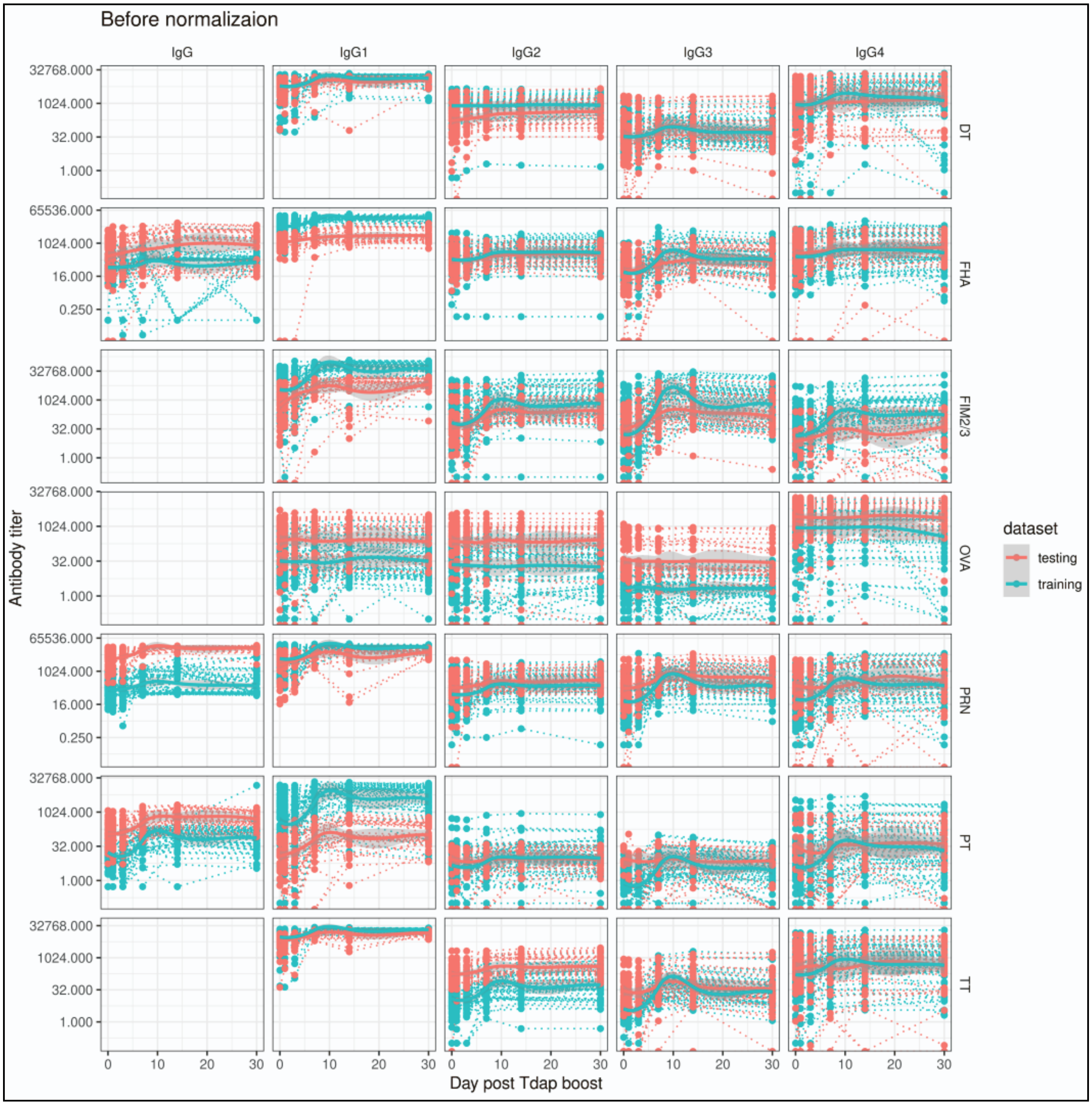

Figure S2: Plot of antibody titer data prior to normalization. Individual plots show the pre- and post-immune response of IgG and its subtypes against antigens. The average titer values differed between the train and test datasets.

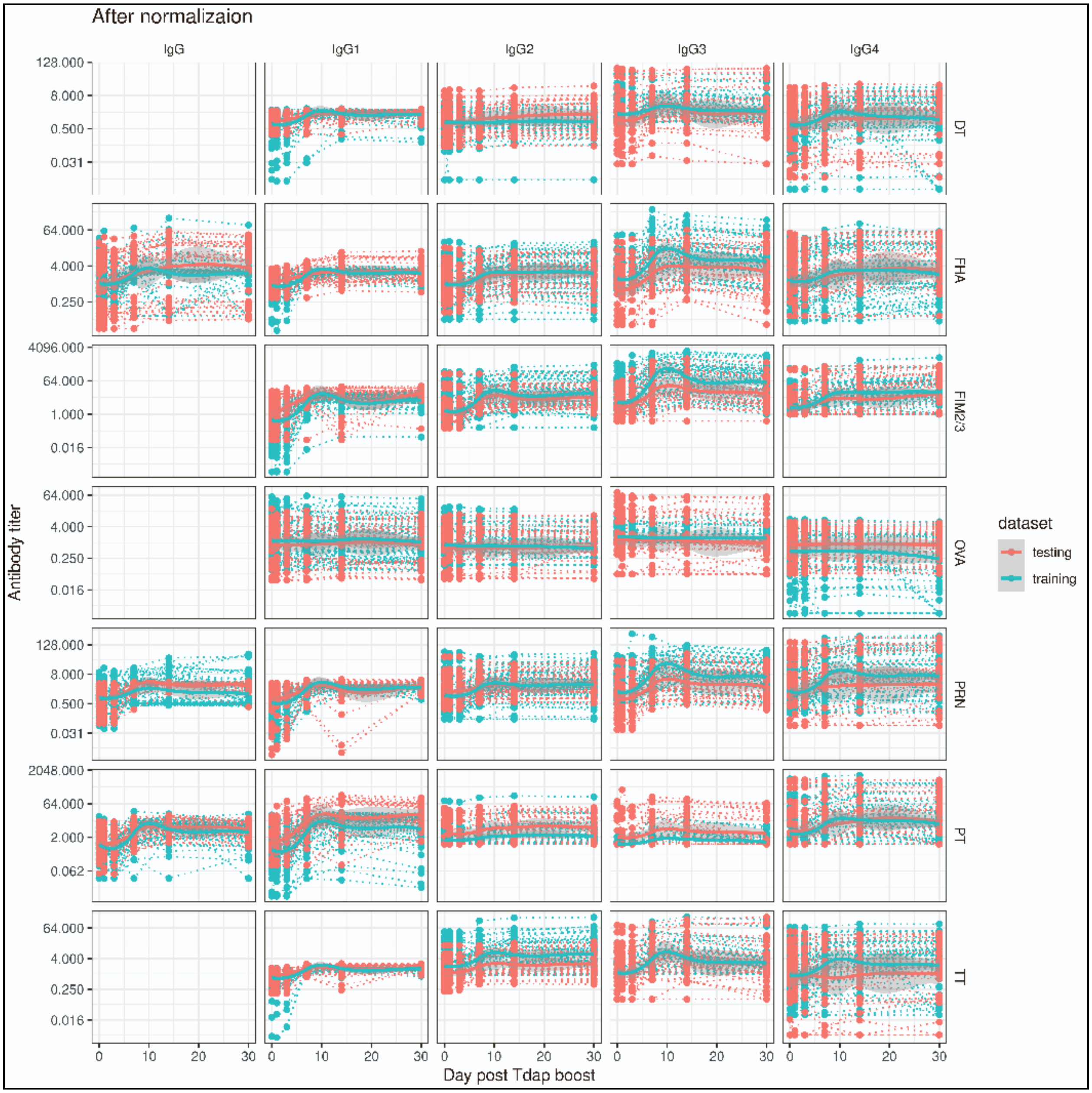

Figure S3: Plot of antibody titer data after normalization. We performed separate standardization of the antibody data in the train and test datasets, using the baseline median as the normalization factor. This approach allowed more direct comparisons of the normalized datasets between the train and test datasets.

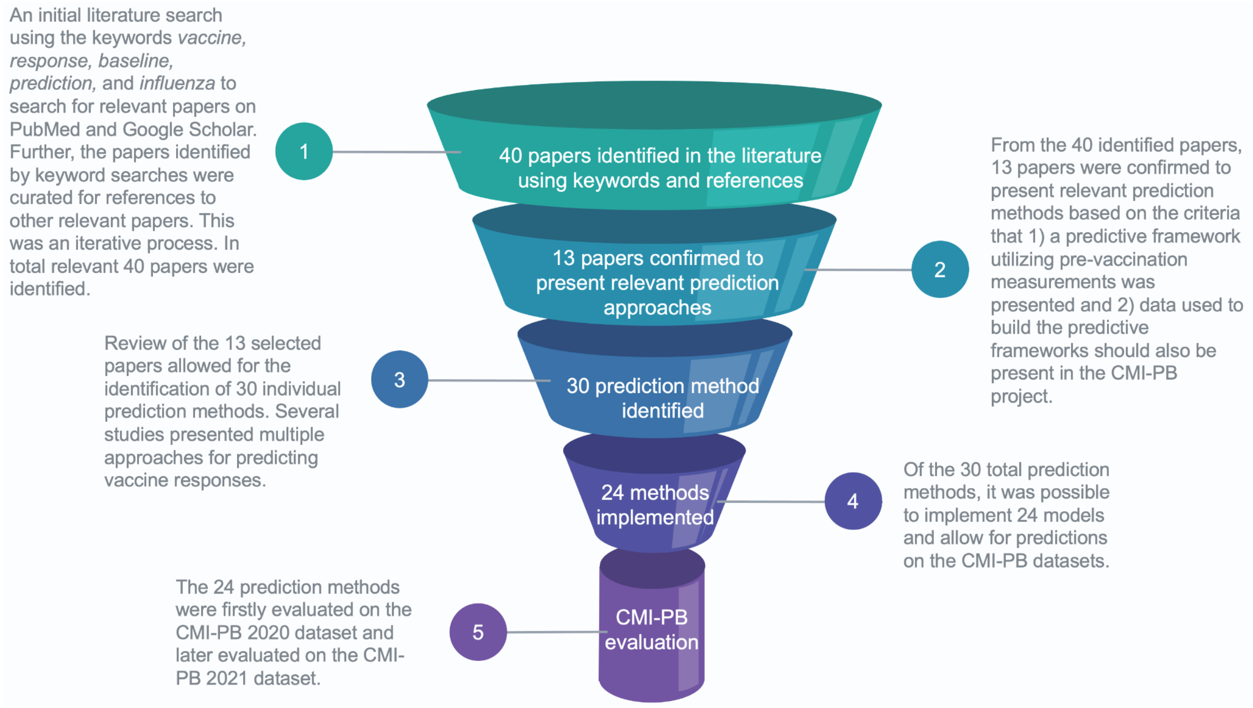

Figure S4: Identification of published methods to predict vaccine responses. The literature search identified previously presented methods for predicting vaccine responses. First, 40 papers were selected from the literature search using keyword searches and relevant references in other papers. From the 40 papers, 13 studies were confirmed to present relevant prediction methods based on a literature search for published vaccine response prediction methods. For several of the 13 relevant studies, multiple prediction methods were presented. In total, 24 prediction methods were implemented from 10 relevant 13 studies (mentioned in **Table S1**). The prediction methods that obtained significant results for both performance metrics in the CMI-PB train datasets were also evaluated in the CMI-PB test dataset used for the first challenge.

Supplementary Tables

Table S1: List of prediction tasks and their biological significance.

	Task Title	Task statement	Biological and clinical significance	Reference
**Antibody level tasks**				
1	IgG-PT_D14	IgG levels against Pertussis Toxin (PT) antigen in plasma on 14 days post booster vaccination	Vaccine-induced immunity for *B. pertussis*	[1]
2	IgG-FHA_D14	IgG levels against Filamentous hemagglutinin (FHA) antigen in plasma on 14 days post booster vaccination	Vaccine-induced immunity for *B. pertussis*	[1]
3	IgG-Pertactin_D14	IgG levels against Pertactin antigen in plasma on 14 days post booster vaccination	Vaccine-induced immunity for *B. pertussis*	Figure 5 in [1]
4	IgG1-PT_D14	IgG1 levels against PT antigen in plasma on 14 days post booster vaccination	Vaccine-induced immunity for *B. pertussis*	Figure 5 in [1]
5	IgG1-FHA_D14	IgG1 levels against FHA antigen in plasma on 14 days post booster vaccination	Vaccine-induced immunity for *B. pertussis*	Figure 5 in [1]
6	IgG4-PT_D14	IgG4 levels against PT antigen in plasma on 14 days post booster vaccination	Signature of Acellular Pertussis (aP) vs. whole-cell Pertussis (wP) response	Figure 5 in [1]
7	IgG4-FHA_D14	IgG4 levels against FHA antigen in plasma on 14 days post booster vaccination	Signature of aP vs. wP response	Figure 5 in [1]1
**Cell frequency tasks**				
8	Plasmablast_D7	Plasmablast cells on day 7 post-booster vaccination	Vaccine-induced immunity for *B. pertussis*	Figure 3 in [1]
9	CD4TCM_D3	CD4 TCM cells on 3 days post booster vaccination	Vaccine-induced immunity for *B. pertussis*	Figure 3 in [1]
10	Monocytes_D3	Monocytes on day 1 post-booster vaccination	Vaccine-induced immunity for *B. pertussis*	Figure 3 in [1]
**Gene expression tasks**				
11	CCL3_D3	CCL3 on day 3 post booster vaccination	Signature of aP vs. wP response	Figure 7 in [1]
12	IL6_D3	IL6 on day 3 post-booster vaccination	Signature of aP vs. wP response	Figure 7 in [1]

13	NFKBIA_D7	NFKBIA at day 7 post booster vaccination	Signature of aP vs. wP response	Figures 6 and 8 in [1]
14	XIST_D14	XIST on day 14 post-booster vaccination	Biological sex-specific marker	[2]

Table S2: Implemented prediction methods for the first challenge. Models 1-24: Supervised methods based on published vaccine response prediction studies. Models 25-43: unsupervised methods based on Multi-omics dimension reduction.

	Model title	Short Description	Method type	Output score	Experimental data	Reference
1	avey_2017_gene_sig	9-gene signature (RAB24, GRB2, DPP3,ACTB, MVP, DPP7, ARPC4, PLEKHB2, and ARRB1)	Classification (Diagonal Linear Discriminant Analysis: DLDA)	geneset signature score	**Experimental data:** PBMC and whole blood gene expression, cell subset frequencies, antibody titers<br><br>**Pathogen:** Influenza virus	Avey et al. 2017 [1]
2	avey_2017_M54	B-cell signaling Blood transcriptome module (BTM; Module M54)	Classification (DLDA)	geneset signature score		
3	avey_2017_M42	Platelet activation (III) (Module M42)	Classification (DLDA)	geneset signature score		
4	avey_2017_M33	Inflammatory response Module (M33)	Classification (DLDA)	geneset signature score		
5	kotliarov_2020_TGSig	10 gene signature (C2orf63, CD101, ENPP1, RETN, SMC1A, ADAM12, EPHB1, PAPSS2,	gene signature score	geneset signature score	**Experimental data:** PBMC and whole blood gene expression, cell subset frequencies, cell surface proteins, antibody titers<br><br>**Pathogen:** Influenza virus, Yellow fever virus	Kotliarov et al. 2020 [2]

		LONP2, C15orf57)				
6	kotliarov_2020_SLE-Sig	Systemic lupus erythematosus (SLE-Sig) gene signature	gene signature score	geneset signature score		
7	kotliarov_2020_IFN-I-DCact	IFN-I-DCact gene signature	gene signature score	geneset signature score		
8	tsang_2014_DLDA_top2	DLDA model (2 cell populations	Classification (DLDA)	Class probabilities	**Experimental data:** PBMC gene expression, cell subset frequencies, antibody titers<br><br>**Pathogen:** Influenza virus	Tsang et al. 2014 [3]
9	tsang_2014_DLDA_top5	DLDA model (5 cell populations)	Classification (DLDA)	Class probabilities		
10	fourati_2015_BioAge	BioAge	Classification (Naïve Bayes)	Class probabilities	**Experimental data:** Whole blood gene expression, cell subset frequencies, serum proteins, antibody titers<br><br>**Pathogen:** Hepatitis B virus	Fourati et al. 2015 [4]
11	fourati_2015_M1	M1 of BioAge	Classification (Naïve Bayes)	Class probabilities		
12	fourati_2015_M1+M16	M1+M16 of BioAge	Classification (Naïve Bayes)	Class probabilities		
13	fourati_2015_NB	Naïve Bayes classifier (15 DEGs)	Classification (Naïve Bayes)	The ratio between the post-probabilities and the log odds in the logistic model		
14	fourati_2015_LR	Logistic regression (4 cell populations)	Regression (logistic)	The ratio between the post-probabilities and the log odds in the logistic model		

15	furman_2013_age	gene signature scores Age	Regression (Elastic net)	age	**Experimental data:** Whole blood gene expression, cell subset frequencies serum cytokines, antibody titers, hemagglutinin peptides<br><br>**Pathogen:** Influenza virus	Furman et al. 2013 [5]
16	iulio_2021_HBV_transfer_sig	HBV pre-vaccine transfer signature	Classification (Random Forest)	geneset signature score	**Experimental data:** PBMC and whole blood gene expression<br><br>**Pathogen:** Influenza virus, Hepatitis B virus, Mycobacterium tuberculosis	Iulio et al. 2021 [6]
17	iulio_2021_Inf_M_transfer_sig	Influenza M pre-vaccine transfer signature	Classification (Random Forest)	geneset signature score		
18	iulio_2021_Inf_F_transfer_sig	Influenza F pre-vaccine transfer signature	Classification (Random Forest)	geneset signature score		
19	iulio_2021_TB_transfer_sig	TB pre-vaccine transfer signature	Classification (Random Forest)	geneset signature score		
20	fourati_2021_RF	Random Forest model (top 500 varying genes)	Classification (Random Forest)	Class probabilities	**Experimental data:** PBMC and whole blood gene expression, cell surface proteins, cell subset frequencies, antibody titers<br><br>**Pathogen:** Influenza, smallpox, Yellow fever virus, Pneumococcal meningococcal	Fourati et al. 2021 [7]
21	bartholomeus_2018_gene_sig	23 differentially expressed genes	Classification (Random Forest)	Class probabilities	**Experimental data:** Whole blood gene expression, absolute numbers of white blood cells, red blood cells,	Bartholomeus et al.

22	bartholomeus_2018_NB	Naïve Bayes classifier (first 5 PCs)	Classification (Naïve Bayes)	Class probabilities	and platelets, antibody titers<br><br>**Pathogen:** Hepatitis B virus	2018 [8]
23	qui_2018_gene_sig	55 up- and 15 down-regulated genes	gene signature score	geneset signature score	**Experimental data:** PBMC gene expression, antibody titers<br><br>**Pathogen:** Hepatitis B virus	Qui et al. 2018 [9]
24	franco_2013_gene_sig	49 genes correlated with antibody response	gene signature score	geneset signature score	**Experimental data:** Whole blood gene expression, whole-genome genotyping, antibody titers<br><br>**Pathogen:** Influenza virus	Franco et al. 2013 [10]
25	jive.elastic_net_cv	Elastic net regression with cross-validation	Regression (Elastic net)		**Experimental data:** PBMC gene expression, cell frequencies, antibody titers	Lock et al. 2013 [11]
26	jive.elastic_net	Elastic net regression	Regression (Elastic net)			
27	jive.lasso_cv	lasso regression with cross-validation	Regression (lasso)			
28	jive.lasso	lasso regression	Regression (lasso)			
29	jive.lr	Linear regression	Regression (linear)			
30	baseline		Regression (lasso)		Baseline tasks, clinical and demographic features	
31	MCIAbasic		Regression (lasso)		**Experimental data:** transcriptome, cell	

					frequencies, antibody levels, cytokines	
32	MCIAplus		Regression (lasso)		Factors from the MCIAbasic model and features from the baseline model	

Table S3. **Summary of questions asked by contestants on the Solutions center.** In total, we received 23 questions that we divided into three categories.

**Category of Questions**	**Number of Questions**	**Summary**
Data access and format	14	Most questions were raised to know details about how contestants can access more data on the analytes' names, how analytes were measured, and the description of the measured unit. Several questions raised issues, like how QC was performed by Olink assay and the role of limit detections on filtering analyte values. Contestants found multiple proteins with duplicate expressions (i.e., IL5). All these questions have been assessed and resolved by the CMI-PB team. Several questions are raised to inquire about difficulties in accessing data API. The CMI-PB team provided direct file download options to contestants. The major concern discussed was antibody titer data was not predictive. Ab titer data normalization was performed using the median at day 0 as a normalization factor for CMI-PB data. Normalization was separately performed on training and test datasets.
Data processing	6	Questions were raised on how to deal with missing values in the data. Missing values arose if analyte measurements were below the limit of the detection threshold and samples needed to be included for subjects to make a complete multi-omics dataset. Out of two that worked with machine learning approaches, one decided to work only with available analyte measurements, and another team worked on data imputation after the decision in a regular meeting.
Miscellaneous (Contest, website)	3	Other questions were specifically asked about how to access challenge tasks, and contestants mentioned that they could not access information about data files on the website.

Table S4. Cohort demographic information

Subject id	Infancy vaccination	Biological sex	Ethnicity	Race	Year of birth	Date of boost
61	wP	Female	Not Hispanic or Latino	Unknown or Not Reported	1/1/1987	4/8/2019
62	wP	Female	Not Hispanic or Latino	Asian	1/1/1993	11/26/2018
63	wP	Female	Not Hispanic or Latino	White	1/1/1995	11/26/2018
64	wP	Male	Not Hispanic or Latino	Asian	1/1/1993	11/26/2018
65	wP	Male	Not Hispanic or Latino	White	1/1/1990	12/3/2018
66	wP	Female	Not Hispanic or Latino	Black or African American	1/1/1976	12/3/2018
67	wP	Female	Hispanic or Latino	White	1/1/1972	1/28/2019
68	wP	Male	Hispanic or Latino	White	1/1/1972	1/28/2019
69	wP	Female	Hispanic or Latino	White	1/1/1990	1/28/2019
70	aP	Male	Not Hispanic or Latino	American Indian/Alaska Native	1/1/1998	1/28/2019
71	aP	Female	Not Hispanic or Latino	White	1/1/1998	1/28/2019
72	wP	Female	Not Hispanic or Latino	White	1/1/1991	2/25/2019
73	wP	Female	Not Hispanic or Latino	White	1/1/1995	2/25/2019
74	wP	Female	Not Hispanic or Latino	White	1/1/1995	2/25/2019
75	aP	Female	Not Hispanic or Latino	Native Hawaiian or Other Pacific Islander	1/1/1998	2/25/2019
76	aP	Female	Not Hispanic or Latino	Asian	1/1/1998	2/25/2019
77	wP	Male	Not Hispanic or Latino	White	1/1/1988	3/18/2019
78	wP	Female	Not Hispanic or Latino	White	1/1/1993	3/18/2019
79	wP	Male	Not Hispanic or Latino	White	1/1/1987	3/18/2019
80	wP	Female	Not Hispanic or	Asian	1/1/1992	3/18/2019

			Latino			
81	wP	Male	Not Hispanic or Latino	White	1/1/1993	3/18/2019
82	aP	Female	Not Hispanic or Latino	More Than One Race	1/1/1998	3/18/2019
83	aP	Female	Not Hispanic or Latino	White	1/1/1999	4/8/2019
84	aP	Female	Not Hispanic or Latino	More Than One Race	1/1/1997	4/8/2019
85	aP	Female	Hispanic or Latino	White	1/1/2000	4/29/2019
86	aP	Female	Not Hispanic or Latino	Asian	1/1/1998	4/29/2019
87	aP	Male	Not Hispanic or Latino	Asian	1/1/2000	4/29/2019
88	aP	Male	Not Hispanic or Latino	Asian	1/1/1900	4/29/2019
89	aP	Female	Not Hispanic or Latino	Asian	1/1/1997	6/3/2019
90	aP	Female	Not Hispanic or Latino	Asian	1/1/1999	6/3/2019
91	aP	Male	Unknown	Unknown or Not Reported	1/1/1998	6/3/2019
92	aP	Female	Hispanic or Latino	White	1/1/2000	6/24/2019
93	aP	Female	Not Hispanic or Latino	More Than One Race	1/1/1996	6/24/2019
94	aP	Male	Not Hispanic or Latino	Unknown or Not Reported	1/1/1999	6/24/2019
95	aP	Female	Hispanic or Latino	Unknown or Not Reported	1/1/1998	6/24/2019
96	aP	Male	Hispanic or Latino	Unknown or Not Reported	1/1/2000	6/24/2019

References:

1. HIPC-CHI Signatures Project Team and HIPC-I Consortium (2017). Multicohort analysis reveals baseline transcriptional predictors of influenza vaccination responses. Sci. Immunol. *2*, eaal4656. 10.1126/sciimmunol.aal4656.
2. Kotliarov, Y., Sparks, R., Martins, A.J., Mulè, M.P., Lu, Y., Goswami, M., Kardava, L., Banchereau, R., Pascual, V., Biancotto, A., et al. (2020). Broad immune activation underlies shared set point signatures for vaccine responsiveness in healthy individuals and disease activity in patients with lupus. Nat Med *26*, 618–629. 10.1038/s41591-020-0769-8.
3. Tsang, J.S., Schwartzberg, P.L., Kotliarov, Y., Biancotto, A., Xie, Z., Germain, R.N., Wang, E., Olnes, M.J., Narayanan, M., Golding, H., et al. (2014). Global analyses of human immune variation reveal baseline predictors of postvaccination responses. Cell *157*, 499–513. 10.1016/j.cell.2014.03.031.
4. Fourati, S., Cristescu, R., Loboda, A., Talla, A., Filali, A., Railkar, R., Schaeffer, A.K., Favre, D., Gagnon, D., Peretz, Y., et al. (2016). Pre-vaccination inflammation and B-cell signalling predict age-related hyporesponse to hepatitis B vaccination. Nat Commun *7*, 10369. 10.1038/ncomms10369.
5. Furman, D., Jojic, V., Kidd, B., Shen-Orr, S., Price, J., Jarrell, J., Tse, T., Huang, H., Lund, P., Maecker, H.T., et al. (2013). Apoptosis and other immune biomarkers predict influenza vaccine responsiveness. Mol Syst Biol *9*, 659. 10.1038/msb.2013.15.
6. di Iulio, J., Bartha, I., Spreafico, R., Virgin, H.W., and Telenti, A. (2021). Transfer transcriptomic signatures for infectious diseases. Proc Natl Acad Sci U A *118*. 10.1073/pnas.2022486118.
7. Fourati, S., Tomalin, L.E., Mulè, M.P., Chawla, D.G., Gerritsen, B., Rychkov, D., Henrich, E., Miller, H.E.R., Hagan, T., Diray-Arce, J., et al. (2022). Pan-vaccine analysis reveals innate immune endotypes predictive of antibody responses to vaccination. Nat Immunol *23*, 1777–1787. 10.1038/s41590-022-01329-5.
8. Bartholomeus, E., De Neuter, N., Meysman, P., Suls, A., Keersmaekers, N., Elias, G., Jansens, H., Hens, N., Smits, E., Van Tendeloo, V., et al. (2018). Transcriptome profiling in blood before and after hepatitis B vaccination shows significant differences in gene expression between responders and non-responders. Vaccine *36*, 6282–6289. 10.1016/j.vaccine.2018.09.001.
9. Qiu, S., He, P., Fang, X., Tong, H., Lv, J., Liu, J., Zhang, L., Zhai, X., Wang, L., Hu, Z., et al. (2018). Significant transcriptome and cytokine changes in hepatitis B vaccine non-responders revealed by genome-wide comparative analysis. Hum Vaccin Immunother *14*, 1763–1772. 10.1080/21645515.2018.1450122.
10. Franco, L.M., Bucasas, K.L., Wells, J.M., Niño, D., Wang, X., Zapata, G.E., Arden, N., Renwick, A., Yu, P., Quarles, J.M., et al. (2013). Integrative genomic analysis of the human immune response to influenza vaccination. Elife *2*, e00299. 10.7554/eLife.00299.
11. Lock, E.F., Hoadley, K.A., Marron, J.S., and Nobel, A.B. (2013). Joint and individual variation explained (jive) for integrated analysis of multiple data types. Ann Appl Stat *7*, 523–542. 10.1214/12-AOAS597.
